# Management of Breast Phyllodes Tumors: A Grade-Stratified Surgical and Reconstructive Strategy

**DOI:** 10.3390/jcm15187226

**Published:** 2026-09-17

**Authors:** Byeongju Kang, Seok Gi Kim, Tae Hwan Park, Jongmoo Park, Sungdae Na, In Hee Lee, Eungbae Lee, Joon Suk Moon, Jeeyeon Lee, Ho Yong Park, Jeong Yeop Ryu, Kang Young Choi, Jung Dug Yang, Ho Yun Chung, Joon Seok Lee

**Affiliations:** 1 Department of Surgery, School of Medicine, Kyungpook National University Chilgok Hospital, Kyungpook National University, Daegu 41404, Republic of Korea; libertas033@knu.ac.kr (B.K.); joonsukm@knu.ac.kr (J.S.M.); j.lee@knu.ac.kr (J.L.); phy123@knu.ac.kr (H.Y.P.); 2 Department of Plastic and Reconstructive Surgery, School of Medicine, Kyungpook National University, Daegu 41566, Republic of Korea; knuhps23090@knuh.kr (S.G.K.); ptw1107@knuh.kr (T.H.P.); prsryu@knu.ac.kr (J.Y.R.); kychoi@knu.ac.kr (K.Y.C.); lambyang@knu.ac.kr (J.D.Y.); hy-chung@knu.ac.kr (H.Y.C.); 3 Department of Radiation Oncology, School of Medicine, Kyungpook National University, Daegu 41566, Republic of Korea; fauny11@naver.com; 4 Department of Biomedical Engineering, Kyungpook National University Hospital, Daegu 41404, Republic of Korea; bluepoison14@knu.ac.kr; 5 Department of Oncology/Hematology, Kyungpook National University Chilgok Hospital, School of Medicine, Kyungpook National University, Daegu 41566, Republic of Korea; ihleeoncology@knu.ac.kr; 6 Department of Thoracic Surgery, School of Medicine, Kyungpook National University, Daegu 41566, Republic of Korea; bay@knu.ac.kr

**Keywords:** phyllodes tumor, breast, histologic grade, mastectomy, breast reconstruction, chest-wall resection

## Abstract

**Background:** Phyllodes tumors (PTs) are rare fibroepithelial breast neoplasms spanning a benign-to-malignant spectrum, and the extent of surgery and reconstruction differs markedly by histologic grade. The primary aim of this study was to derive a grade-stratified surgical and reconstructive algorithm. **Methods:** The charts of patients diagnosed with phyllodes tumor at Kyungpook National University Chilgok Hospital between January 2011 and February 2026 were retrospectively reviewed. Tumors were classified by World Health Organization criteria as benign, borderline, or malignant, and the treatment protocols, extent of surgery, and immediate reconstruction after mastectomy were analyzed. **Results:** Of 417 patients charted, 401 were confirmed to have phyllodes tumors and 334 carried a definitive histologic grade. Together, they formed the analyzed cohort: 251 benign (75.1%), 55 borderline (16.5%), and 28 malignant (8.4%). Benign tumors were managed predominantly by breast-conserving surgery (BCS). Twenty-six patients formed the surgical cohort, defined by the reconstructive requirement of the resection defect: all total mastectomies (n = 19) together with all breast-conserving resections reconstructed by the plastic surgical team (oncoplastic breast-conserving surgery, n = 7). Reconstruction was performed in 21 (80.8%) with latissimus dorsi-based flaps, local flaps, or direct-to-implant reconstruction. Adjuvant radiotherapy was confined to malignant disease (7 of 15 malignant PTs, 46.7%; *p* = 0.010). Over a median follow-up of 32.7 months, local recurrence developed in four patients (15.4%) and two malignant tumors were fatal. In an illustrative case of recurrent malignant PT with rib metastasis, a widely opened pleural cavity was sealed with a single sheet of non-fenestrated acellular dermal matrix (ADM), which was found to be fully incorporated and still sealing the pleura at later implant explantation. **Conclusions:** A grade-stratified strategy (BCS for benign tumors and mastectomy with reconstruction for borderline/malignant disease) balances oncologic safety with aesthetic outcome.

## 1. Introduction

Phyllodes tumors (PTs) are rare fibroepithelial neoplasms of the breast, accounting for fewer than 1% of all breast tumors [1]. Although they share the biphasic stromal–epithelial architecture of fibroadenomas, they are distinguished by a characteristic leaf-like growth pattern and a cellular stroma. According to the current (fifth edition, 2019) World Health Organization (WHO) classification, PTs are graded as benign, borderline, or malignant on the basis of tumor margins, stromal cellularity, stromal atypia, mitotic activity, stromal overgrowth, and the presence of heterologous elements [1,2]. This histologic grading is not merely descriptive: it predicts the risk of local recurrence and distant metastasis and guides the extent of treatment [2,3].

Clinically, most PTs present as a rapidly enlarging, painless, palpable breast mass and appear as a well-circumscribed lesion on mammography and ultrasonography, closely resembling a fibroadenoma and rendering preoperative diagnosis difficult. Core needle biopsy has a substantial false-negative rate for distinguishing PT from fibroadenoma; accordingly, in a rapidly growing “giant fibroadenoma-like” mass, the possibility of a PT should always be considered, and excision with an adequate margin should be pursued [4]. Importantly, core needle biopsy fails to exclude a PT and reliably assigns the benign, borderline, or malignant grade in only a minority of cases; in most fibroepithelial lesions, the histologic grade cannot be determined preoperatively, so an excisional specimen is more often than not required for definitive grading.

Although complete surgical excision with negative margins is the basic principle of treatment, the required extent of resection and the need for adjuvant therapy differ substantially by histologic grade. Benign PTs can frequently be managed by breast-conserving surgery (BCS), with a generally favorable prognosis once negative margins are achieved, whereas inadequate margins are associated with local recurrence even in benign lesions [2,3].

In contrast, borderline and malignant PTs behave in a sarcoma-like fashion, with high stromal cellularity, marked atypia, and infiltrative growth; approximately 20% of malignant tumors develop hematogenous metastases, most often to the lungs and bones [5,6,7]. For these grades a wider margin is sought, and total mastectomy, occasionally including full-thickness chest-wall resection with immediate reconstruction, is required for tumors that are large, multifocal, recurrent, or invading the chest wall [8,9]. As axillary nodal metastasis is rare, routine axillary dissection is not recommended, and systemic chemotherapy or endocrine therapy has a very limited role [5].

More recently, nipple-sparing mastectomy with immediate breast reconstruction has been applied to malignant PT with maintained oncologic safety and satisfactory cosmetic outcomes if the nipple–areola complex is uninvolved and the retroareolar margin is negative [10,11,12,13].

In this context, the primary objective of the present study was to derive and present a grade-stratified surgical and reconstructive algorithm for phyllodes tumor. Grade-specific principles for determining the extent of resection, the application of total mastectomy with immediate reconstruction for malignant PT, and reconstructive strategies for chest-wall invasion are summarized and presented as an institutional treatment algorithm and a reconstruction algorithm.

## 2. Materials and Methods

### 2.1. Study Design and Population

A retrospective review was conducted of patients diagnosed and treated for phyllodes tumor of the breast at Chilgok Kyungpook National University Hospital between January 2011 and February 2026. All cases charted with a diagnosis of phyllodes tumor during this period were identified from the institutional pathology database. Specimens were classified according to the WHO criteria as benign, borderline, or malignant PT; needle-biopsy impressions in which a fibroepithelial lesion could not be reliably distinguished from fibroadenoma were recorded as indeterminate. For each patient, the diagnosis, the specimen or operative method, and, where applicable, the type of mastectomy and reconstruction were recorded. The study was approved by the Institutional Review Board of Kyungpook National University Chilgok Hospital (IRB File No. KNUCH 2026-03-060-002), and the requirement for informed consent was waived owing to the retrospective nature of the study. All procedures were performed in accordance with the ethical standards of the institutional research committee and with the 1964 Declaration of Helsinki and its later amendments.

Two cohorts were defined. The whole cohort comprised every phyllodes tumor identified in the pathology database and was used only to establish the grade distribution and the overall pattern of surgical management. The surgical cohort, which is the subject of the outcome analyses and of the algorithm presented here, comprised every patient who underwent total mastectomy, or a breast-conserving resection requiring reconstruction by the plastic surgical team, for a phyllodes tumor and for whom the operative record, the definitive histopathological report and at least one follow-up assessment could all be retrieved; patients whose operative or pathological documentation was incomplete were excluded from this cohort, although they remain in the whole-cohort denominator. Receipt of reconstruction was not an inclusion criterion.

The surgical cohort is defined by the reconstructive requirement of the resection defect. It comprises every patient who underwent total mastectomy (n = 19), together with every patient whose breast-conserving resection produced a defect that could not be closed directly and was reconstructed by the plastic surgical team at the same operation (oncoplastic breast-conserving surgery, n = 7). Conserving resections closed directly, whether recorded as breast-conserving surgery or as partial mastectomy in our operative records, are not part of this cohort and are counted in the whole-cohort denominator only. It therefore corresponds to the population in whom a reconstructive decision arose, which is the population to which the algorithm presented here applies. Within it, reconstruction was performed in 21 of 26 patients (80.8%); the 5 patients closed without reconstruction had all undergone total mastectomy.

### 2.2. Treatment Protocol: Benign Phyllodes Tumor

Breast imaging (mammography and ultrasonography) was performed in all patients, and core needle biopsy was used to confirm a fibroepithelial lesion or suspected PT. For lesions diagnosed as, or strongly suspicious for, benign PT, therapeutic excision (excisional biopsy or BCS) was performed as a first-line treatment, aiming for complete removal of the tumor together with its capsule while minimizing breast deformity. An intact capsule with microscopically negative (ink-negative) margins, rather than a fixed 1 cm gross margin as advised by guidelines for benign tumors [14], was considered adequate, sparing the patient further wide re-excision. A positive margin was not routinely re-excised unless the specimen was transected or gross residual disease was suspected. Cases of confirmed benign PT with negative margins were followed without adjuvant radiotherapy or chemotherapy, with physical examination and ultrasonography (mammography as needed) every 6–12 months for the first 2–3 years and annually thereafter. Suspected local recurrence was confirmed by imaging and biopsy; recurrent benign PT was treated by repeat BCS, with total mastectomy reserved for very large or repeatedly recurrent lesions unsuitable for breast preservation.

### 2.3. Treatment Protocol: Borderline and Malignant Phyllodes Tumor

**Figure 1:**
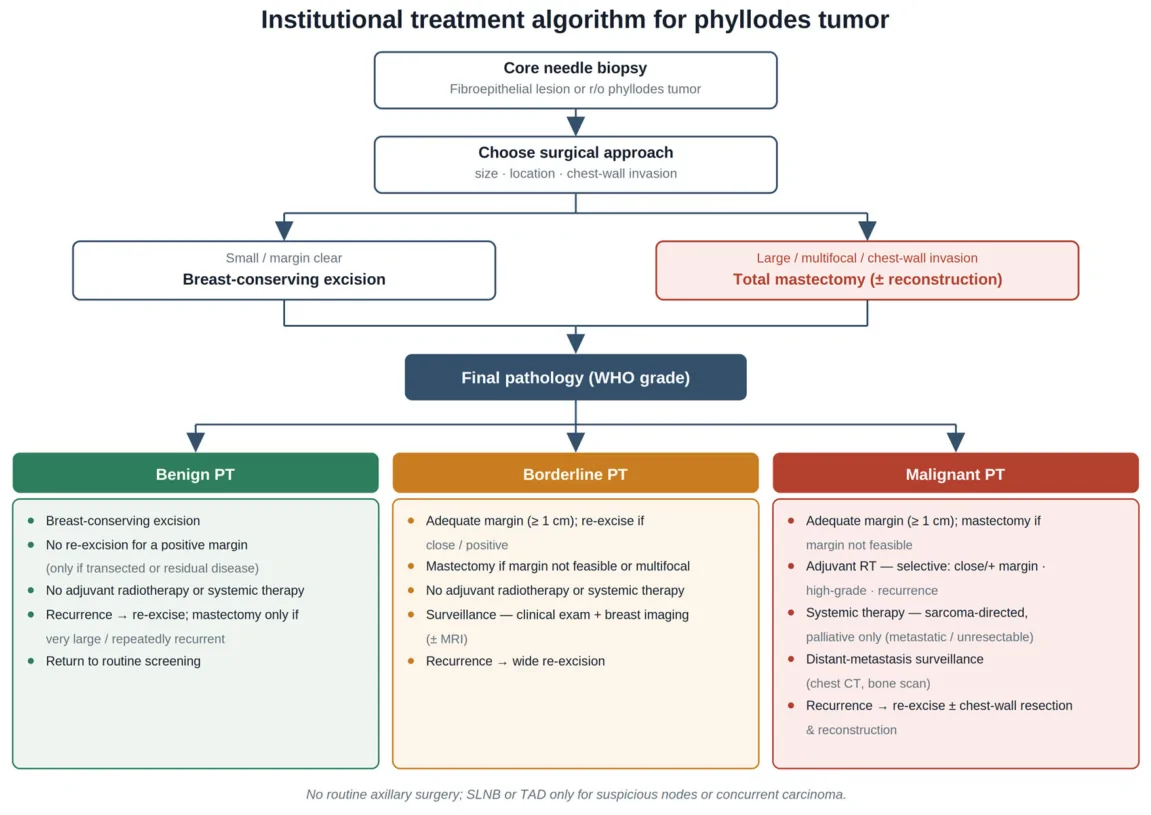
Institutional treatment algorithm for breast phyllodes tumors. RT—radiotherapy.

Lesions classified as borderline or malignant PT (collectively termed non-benign) were evaluated with mammography, ultrasonography, and, as indicated, breast magnetic resonance imaging to assess tumor size, location, and skin or chest-wall involvement. Given the rapid growth and the risk of local recurrence and distant metastasis, surgery was planned promptly. The extent of resection was determined by tumor size, location, and the tumor-to-breast ratio. For relatively small tumors in which an adequate margin could be obtained without unacceptable cosmetic or functional compromise, wide local excision with a gross margin of at least 1 cm was performed. For tumors that were large, multifocal, or recurrent, with otherwise unavoidable breast deformity, or with suspected chest-wall invasion, total mastectomy was selected as the primary procedure. This grade-stratified escalation of surgical extent, together with the indications for adjuvant therapy, is summarized in the institutional treatment algorithm (Figure 1). As axillary nodal metastasis is rare, routine axillary surgery was avoided; sentinel lymph node biopsy (SLNB) or targeted axillary dissection (TAD) were selectively performed in patients with clinically suspicious nodes or concurrent carcinoma. In patients undergoing total mastectomy, immediate breast reconstruction was planned where feasible, and positive or markedly close margins were managed by re-excision on an individualized basis. Adjuvant therapy was grade-dependent: borderline tumors received no adjuvant treatment, whereas malignant tumors were considered for selective adjuvant radiotherapy and, for metastatic or unresectable disease, palliative sarcoma-directed systemic therapy (detailed below). Systematic follow-up was undertaken for all patients, with history and physical examination every 3–6 months for the first 2–3 years, every 6–12 months until 5 years, and annually thereafter. For borderline tumors, surveillance imaging comprised mammography and ultrasonography, supplemented by breast magnetic resonance imaging as indicated; for malignant tumors, chest computed tomography and bone scintigraphy were additionally performed to screen for distant metastasis. Local recurrence of a borderline tumor was treated by wide re-excision with repeat reconstruction, whereas recurrence of a malignant tumor was managed by re-excision or, when the chest wall was involved, en bloc chest-wall resection with reconstruction; distant metastasis was managed with a multidisciplinary palliative approach.

### 2.4. Reconstruction Technique

Reconstructive procedures are reported by reconstructive intent, as breast mound reconstruction or as chest-wall and soft-tissue coverage, and are not pooled in any analysis.

*Mastectomy:* In patients with malignant PT, total mastectomy was performed under general anesthesia with the patient positioned supine and slightly elevated; the skin incision was designed according to tumor size and location and the presence of skin or chest-wall involvement. For localized tumors lying at a sufficient distance from the nipple–areola complex (NAC), skin-sparing or nipple-sparing mastectomy was preferred within the limits of oncologic safety, whereas simple mastectomy was performed for tumors abutting the NAC or involving the skin. The breast was removed en bloc with a uniform flap thickness, including the pectoralis fascia deep to the tumor. For a planned nipple-sparing mastectomy, intraoperative frozen-section examination of the retroareolar tissue was performed, and the NAC was excised if the margin proved positive.

**Figure 2:**
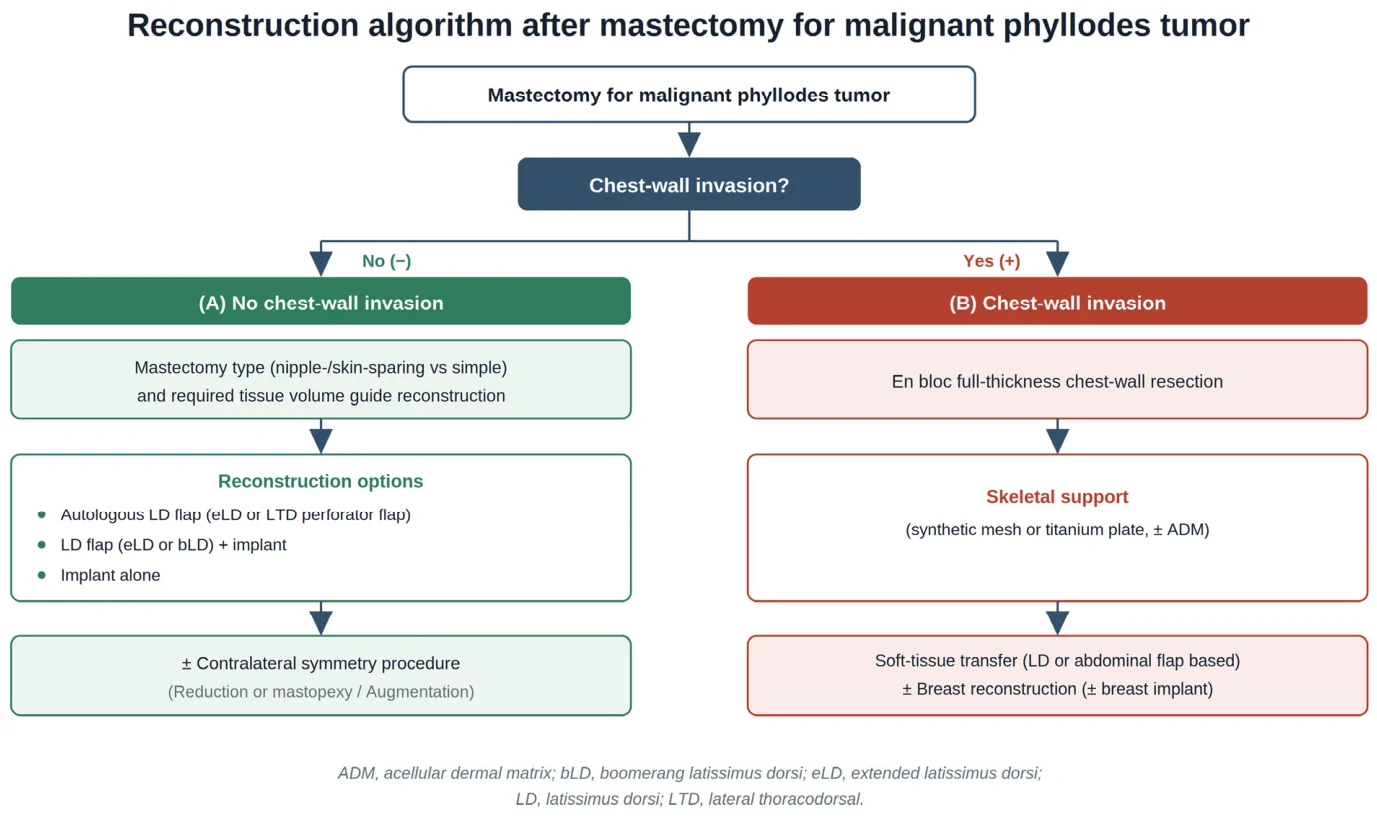
Reconstruction algorithm after mastectomy for malignant breast phyllodes tumor.

*Reconstructive planning:* Immediate reconstruction was performed at the same operative setting as the mastectomy. The technique was individualized according to body habitus, contralateral breast volume and shape, chest-wall and pectoralis status, comorbidities, prior or planned radiotherapy, and patient preference. The available options are summarized in Figure 2(A).

*Latissimus dorsi flap-based breast reconstruction:* All autologous and implant-assisted reconstructions were LD-based, with the design varying with the size of the skin defect and the breast volume to be restored. The muscle was harvested as a myocutaneous flap through an oblique dorsal incision, elevated on its thoracodorsal pedicle, passed through the axillary tunnel and inset to recreate the breast mound, with the donor site closed primarily over a closed-suction drain. For a very large skin defect, the paddle was designed in a boomerang (angled, V-shaped) configuration (bLD), which recruits additional skin while permitting a tension-free linear donor-site closure, and was combined with a submuscular implant. For a smaller defect, the choice followed breast size: an extended LD (eLD) flap alone, raising the paddle with a cuff of subcutaneous fat, where the breast was small and a prosthesis was to be avoided; or an eLD flap with a submuscular implant for a moderate-to-large breast, which also provides vascularized coverage over the prosthesis where radiotherapy is anticipated.

*Implant-based breast reconstruction:* For implant-only reconstruction, a subpectoral or dual-plane pocket was created, and the inferolateral pole was reinforced with acellular dermal matrix (ADM) where additional lower-pole support was required. This single-stage, direct-to-implant approach was reserved for patients with an uninvolved pectoralis, adequate and well-perfused mastectomy skin flaps, and no planned adjuvant radiotherapy.

*Chest-wall reconstruction:* For tumors invading the chest wall and requiring resection of muscle, ribs, or pleura, the lesion and the involved structures were resected en bloc. Skeletal stability was restored with synthetic mesh or a titanium plate, supplemented by ADM where indicated to seal the pleural cavity, and the repair was resurfaced with a latissimus dorsi flap or, for more extensive defects, an alternative myocutaneous or pedicled flap (e.g., transverse rectus abdominis myocutaneous [TRAM] or omental flap) to provide durable, vascularized soft-tissue coverage (Figure 2(B)). Where the deficiency was confined to the lateral chest wall, a lateral thoracodorsal (LTD) perforator flap was raised from the lateral thoracic region and rotated medially, preserving the LD muscle and its function.

*Symmetry and postoperative care:* A contralateral symmetry procedure (reduction, mastopexy, or augmentation) was performed concurrently or in a staged fashion where indicated. Closed-suction drains were placed; the wounds were closed in layers without tension; and flap perfusion, implant position, and wound healing were monitored postoperatively, followed by progressive rehabilitation of shoulder and arm function.

### 2.5. Adjuvant Radiotherapy

Adjuvant radiotherapy was administered to patients with high-risk features, including close or positive margins and large or high-grade tumors. The target was the tumor bed after BCS, or the reconstructed breast or chest wall after mastectomy, and regional nodal irradiation was added only in patients with nodal involvement. A boost to the tumor bed or chest-wall scar was added for close or positive margins. The prescribed dose was 50 Gy in 25 fractions or 40–42.6 Gy in 15–16 fractions to the breast or chest wall, with a boost of 10–16 Gy in 4–8 fractions where indicated; fractionation shifted from conventional to hypofractionated over the study period and thus varied by treatment era. If an invasive or in situ carcinoma coexisted with the phyllodes tumor, the indication, target volume (including regional nodal treatment), and use of a boost were governed by the carcinoma rather than the phyllodes tumor.

### 2.6. Systemic Therapy

Systemic chemotherapy was withheld as adjuvant treatment for localized disease. Chemotherapy was reserved for the palliative treatment of metastatic or unresectable disease and, because malignant PTs behave like soft-tissue sarcomas, followed sarcoma-type regimens (e.g., doxorubicin- and ifosfamide-based), applied after multidisciplinary discussion [7,8,15,16]. The decision to initiate systemic therapy, and the choice of regimen, were individualized according to disease burden, resectability of metastases, and performance status. Endocrine therapy had no established role. Targeted therapies and immunotherapies are also not recommended, and these areas warrant further research.

### 2.7. Statistical Analysis

Categorical variables are summarized as counts and percentages, and continuous variables as the mean ± standard deviation (age and body mass index) or the median and range (follow-up, radiotherapy dose, and the intervals from operation to recurrence and to last contact). Lesions with an indeterminate needle-biopsy impression (“r/o phyllodes”) or without a stated histologic grade were excluded from the graded analysis, no grade being imputed; age and body mass index were compared across grades by one-way analysis of variance. Oncologic outcomes were analyzed in the 26 patients of the surgical cohort. Associations of histologic grade with receipt of adjuvant radiotherapy and of chemotherapy, and of histologic grade, extent of surgery, adjuvant radiotherapy, and chemotherapy with local recurrence, were assessed with the Fisher’s exact test (malignant vs. benign or borderline tumors for grade). As recurrence, death, and irradiation events were few, all outcome analyses are exploratory and the reported *p* values are descriptive; a two-sided *p* value < 0.05 was considered significant. Analyses were performed with SPSS Statistics version 27.0 (IBM Corp., Armonk, NY, USA), and the study is reported in accordance with the STROBE (Strengthening the Reporting of Observational Studies in Epidemiology) statement.

## 3. Results

### 3.1. Histologic Classification

**Figure 3:**
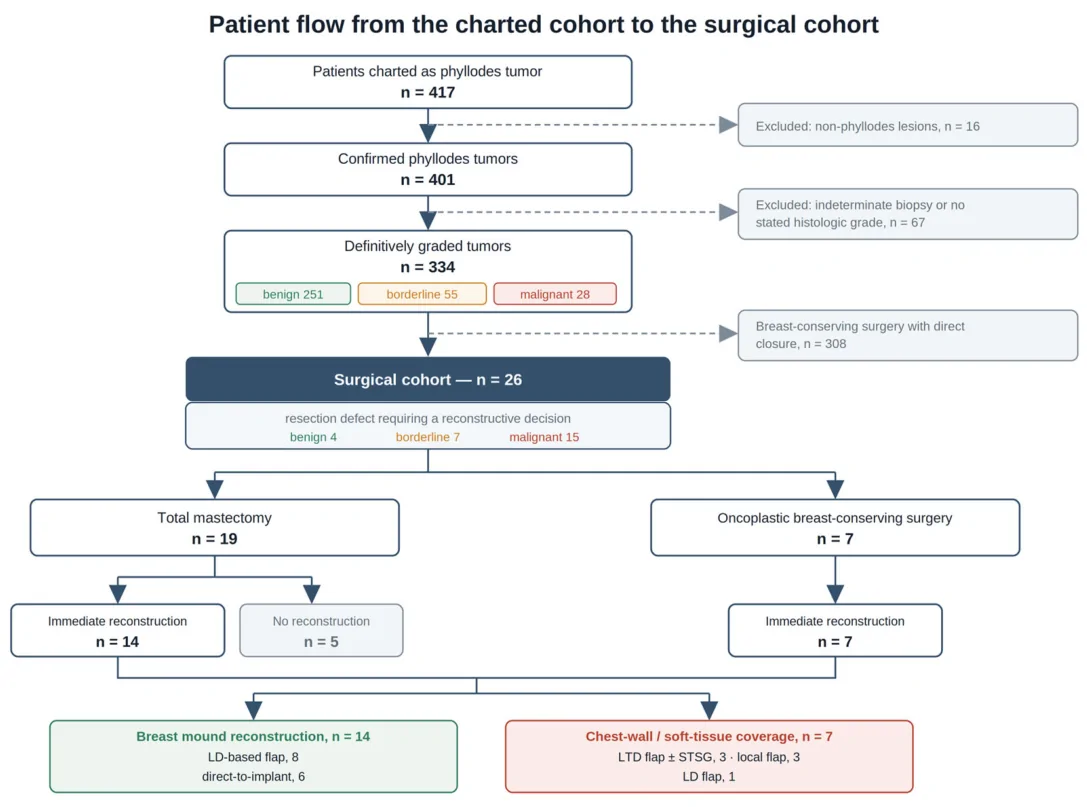
Flow of patients from the charted cohort to the surgical cohort and its reconstructive subsets. BCS—breast-conserving surgery; DTI—direct-to-implant; LD—latissimus dorsi; LTD—lateral thoracodorsal; STSG—split-thickness skin graft.

Of 417 patients charted with a phyllodes tumor between 2011 and 2026, 16 were excluded as non-phyllodes lesions, leaving 401 confirmed phyllodes tumors. A further 67 lesions carried an indeterminate needle-biopsy impression (a fibroepithelial lesion in which fibroadenoma and PT could not be reliably distinguished) or lacked a stated histologic grade; because grade determines every subsequent treatment decision in this study, these were excluded from the graded analysis rather than assigned a grade by imputation, leaving 334 definitively graded tumors as the analyzed cohort. Of these, 251 (75.1%) were benign, 55 (16.5%) borderline, and 28 (8.4%) malignant; borderline and malignant tumors, collectively termed non-benign, accounted for 83 (24.9%) (Table 1, Figure 3). None of the 67 ungraded lesions underwent mastectomy, so their exclusion does not affect the surgical cohort described below. The mean age was higher in borderline and malignant tumors than in benign tumors (one-way ANOVA *p* = 0.017), whereas the body mass index was similar across grades (*p* = 0.411; Table 1).

### 3.2. Surgical Management and Reconstruction

Benign tumors were predominantly managed by BCS, whereas borderline and malignant tumors were treated by BCS, oncoplastic BCS, or total mastectomy according to the protocol described above. Twenty-six patients met the criteria for the surgical cohort defined in Section 2.1, comprising 19 total mastectomies and seven oncoplastic breast-conserving resections (Table 2); the cohort is defined by the reconstructive requirement of the resection defect and by completeness of documentation, and forms the basis for the outcome analyses (Table 3 and Table 4). Reconstruction was performed in 21 of the 26 (80.8%): 14 of the 19 total mastectomies and all seven oncoplastic resections. The five patients closed without reconstruction had all undergone total mastectomy. By histologic grade, the cohort comprised four benign, seven borderline, and 15 malignant tumors; the benign lesions entered the cohort because a large or recurrent tumor required mastectomy or a reconstructed conserving resection. Among the 22 borderline or malignant tumors, 16 underwent total mastectomy and six underwent an oncoplastic conserving resection. Among the 19 total mastectomies, immediate reconstruction was undertaken in 14 (73.7%) when the patient wished it and no oncologic contraindication was present; the remaining five were closed without reconstruction. All seven oncoplastic conserving resections were reconstructed, with reconstruction being intrinsic to the procedure. Procedures are reported by reconstructive intent (Section 2.4). Breast mound reconstruction was performed with latissimus dorsi-based flaps (extended, boomerang or vertical) or direct-to-implant reconstruction, and chest-wall or soft-tissue coverage with local advancement or rotation flaps, with or without split-thickness skin grafting, or full-thickness chest-wall reconstruction (Table 2). The two categories are described separately and are not combined into a single reconstruction group.

**Figure 4:**
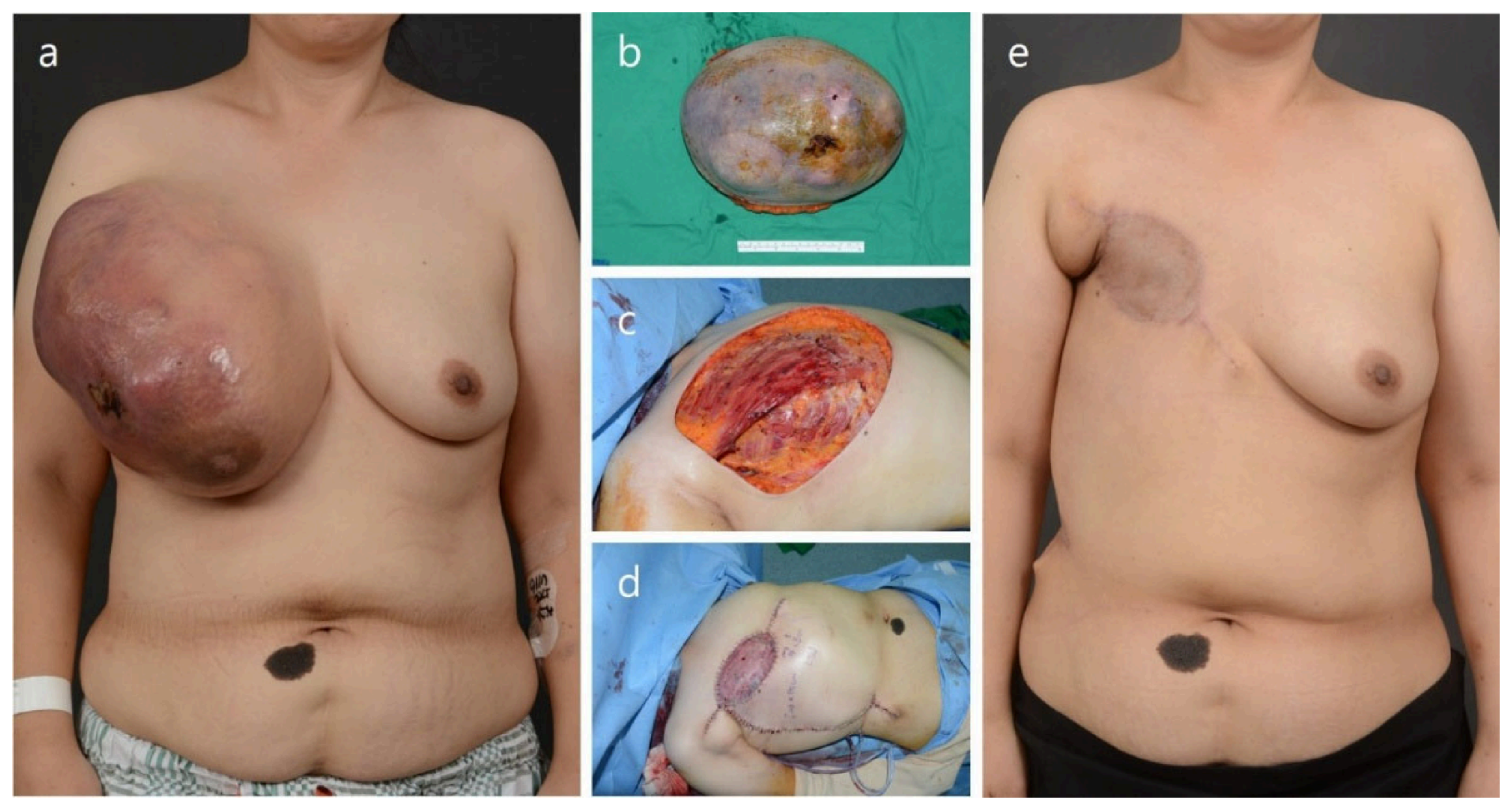
Mastectomy and immediate chest-wall reconstruction for a huge malignant phyllodes tumor. (**a**) Preoperative view of a huge phyllodes tumor on the right breast. (**b**–**d**) Intraoperative photographs showing the excised mass, the resulting chest-wall defect, and immediate reconstruction with a local advancement flap combined with a split-thickness skin graft (STSG). (**e**) Postoperative appearance at 1 month. STSG—split-thickness skin graft.

**Figure 5:**
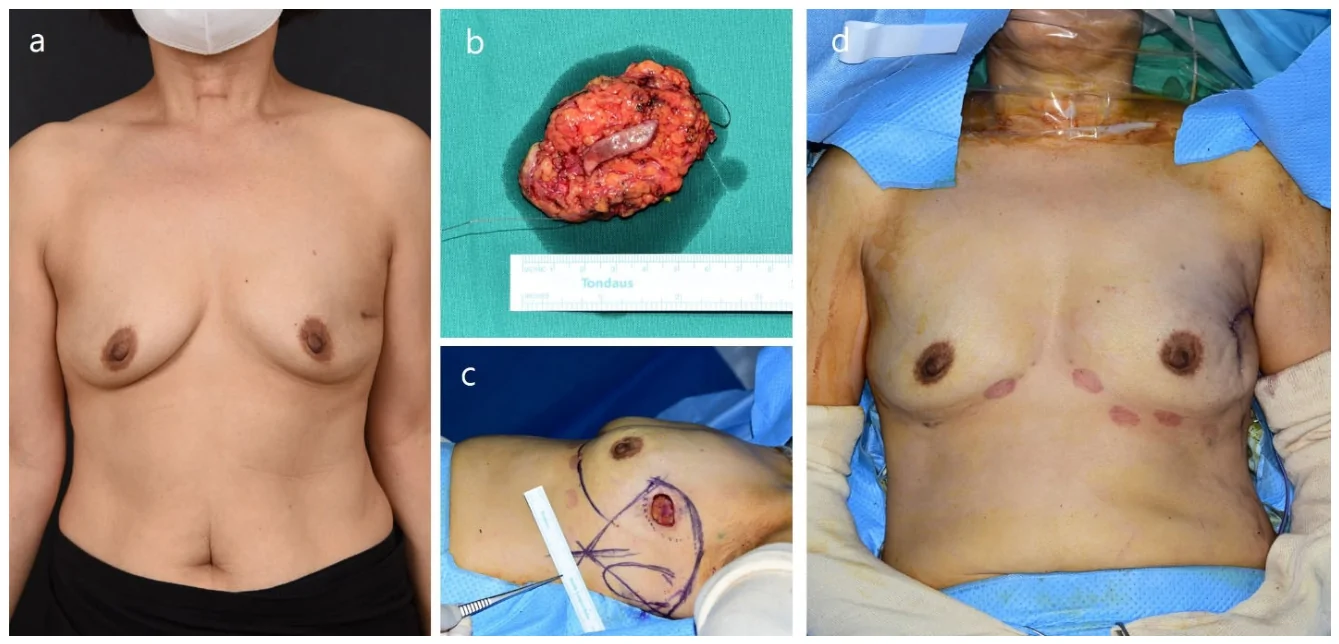
Mastectomy and immediate reconstruction for a moderate-sized malignant phyllodes tumor. (**a**) Preoperative view of a tumor in the lateral aspect of the left breast. (**b**,**c**) Intraoperative photographs showing the excised mass, the resulting defect, and the design of a local rotation flap for reconstruction. (**d**) Immediate postoperative appearance.

**Figure 6:**
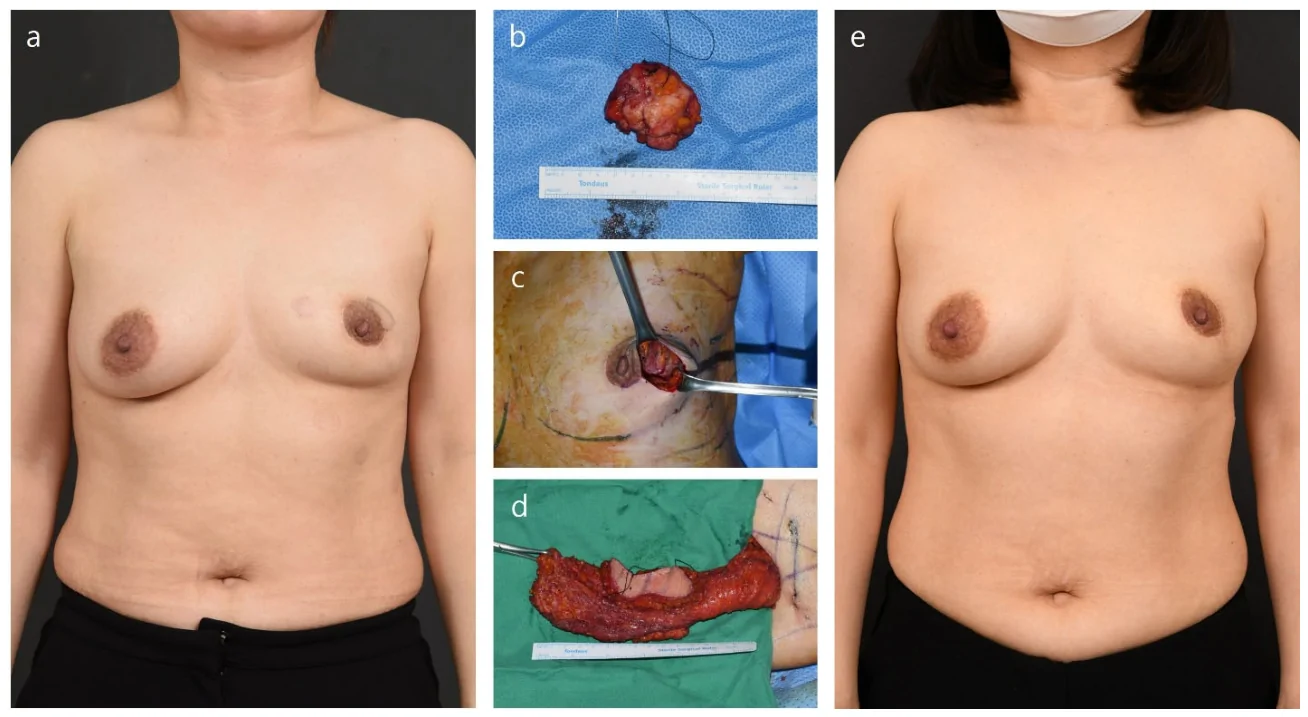
Mastectomy and immediate reconstruction for a small malignant phyllodes tumor. (**a**) Preoperative view of a tumor in the periareolar area of the left breast. (**b**–**d**) Intraoperative photographs showing the excised mass, the resulting defect, and reconstruction with a vertical latissimus dorsi (LD) flap; autologous tissue transfer was chosen for reconstruction and volume correction because the affected left breast was smaller than the contralateral side. (**e**) Postoperative appearance at 2 weeks. LD—latissimus dorsi.

Representative cases illustrating mastectomy and immediate reconstruction for malignant phyllodes tumors across the spectrum of tumor burden are shown in Figure 4, Figure 5 and Figure 6: a large tumor requiring chest-wall coverage with a local advancement flap and split-thickness skin graft (Figure 4), a moderate-sized lesion reconstructed with a local rotation flap (Figure 5), and a small periareolar tumor reconstructed with a vertical latissimus dorsi flap (Figure 6).

### 3.3. Adjuvant Radiotherapy

Among the 26 patients of the surgical cohort, adjuvant radiotherapy was administered to seven (26.9%), all for the phyllodes tumor. Phyllodes-directed radiotherapy was confined to malignant tumors (seven of 15 malignant tumors [46.7%] vs. 0 of 11 benign or borderline; Fisher’s exact *p* = 0.010; Table 3). Detail was available for six of the seven phyllodes-directed courses, with one course having been delivered at an outside hospital: conventional fractionation was used in four and hypofractionation in two, a tumor-bed boost was added in three, and the median total dose was 52.6 Gy (range, 50.0–60.0); the RT field is detailed in Table 3. Local recurrence did not differ by receipt of phyllodes-directed radiotherapy (two of seven irradiated vs. two of 19 non-irradiated; Fisher’s exact *p* = 0.287; Table 4).

### 3.4. Recurrence, Mortality, and Systemic Therapy

Over a median follow-up of 32.7 months (range, 0.1–99.5), local recurrence developed in four of the 26 patients (15.4%). Recurrence increased across histologic grade (0 of four benign tumors [0%], one of seven borderline tumors [14.3%], and three of 15 malignant tumors [20.0%]), although the difference between malignant and benign or borderline disease did not reach significance in this small cohort (Fisher’s exact *p* = 0.614; Table 4). By type of resection, recurrence occurred in two of 19 total-mastectomy patients (10.5%) and in two of seven oncoplastic conserving resections (28.6%; Fisher’s exact *p* = 0.287). The median interval from operation to recurrence was 7.1 months (range, 2.7–12.9). Two patients died (7.7%), both of whom had a malignant tumor that had recurred (Table 4).

Adjuvant or palliative chemotherapy was administered to six patients (23.1%), all of whom had a malignant phyllodes tumor (six of 15 malignant tumors, 40.0%; Fisher’s exact *p* = 0.024); no benign or borderline tumor was treated systemically. Reflecting the soft-tissue-sarcoma behavior of high-grade disease, the regimens were sarcoma-directed: doxorubicin alone in three patients and doxorubicin combined with ifosfamide in three. Chemotherapy was associated with local recurrence on univariable testing (three of six treated patients vs. one of 20 untreated patients; Fisher’s exact *p* = 0.028; odds ratio, 19.0; Table 4). Among the treated patients, the duration of chemotherapy ranged from 1.7 to 9.5 months (median, 2.3).

### 3.5. Illustrative Case: Recurrent Malignant Phyllodes Tumor with Rib Metastasis

**Figure 7:**
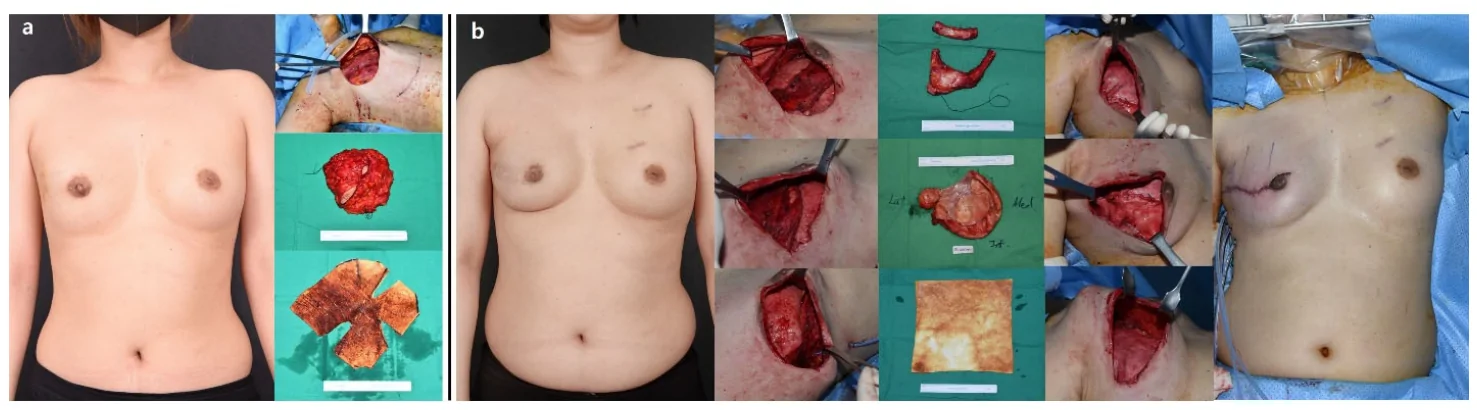
Clinical course of a malignant phyllodes tumor treated by mastectomy with direct-to-implant (DTI) reconstruction and subsequent chest-wall recurrence. (**a**) Primary presentation, post-mastectomy defect, excised specimen and acellular dermal matrix (ADM) implant coverage. (**b**) En bloc resection of the recurrence with the pleural cavity opened and the lung exposed; the reconstructive sequence is shown in Figure 8. ADM—acellular dermal matrix; DTI—direct-to-implant.

An isolated chest-wall recurrence at the costal cartilage of the second, fourth, and fifth ribs developed in a patient previously treated with nipple-sparing mastectomy with direct-to-implant (DTI) reconstruction for a primary malignant phyllodes tumor. Cross-sectional restaging with positron emission tomography–computed tomography (PET/CT) revealed intense hypermetabolic uptake at the costal cartilage, confirming an isolated chest-wall recurrence with rib involvement (Figure S1). As the disease was confined to the chest wall and was judged as resectable, en bloc full-thickness chest-wall resection with immediate reconstruction was undertaken; the resection opened the pleural cavity and exposed the underlying lung (Figure 7).

Histopathological examination of the resected rib confirmed recurrent malignant phyllodes tumor with a purely sarcomatous appearance, the biphasic leaf-like pattern of the primary lesion having been lost; the detailed microscopic findings are given in the legend of Supplementary Figure S2.

Targeted next-generation sequencing of the recurrent lesion identified MED12, TP53 and NRAS mutations with a chromosome 8q gain, a low tumor mutational burden and a microsatellite-stable, genomically stable profile. No clinically actionable alteration was found; the profile and its implications are considered in the Discussion.

**Figure 8:**
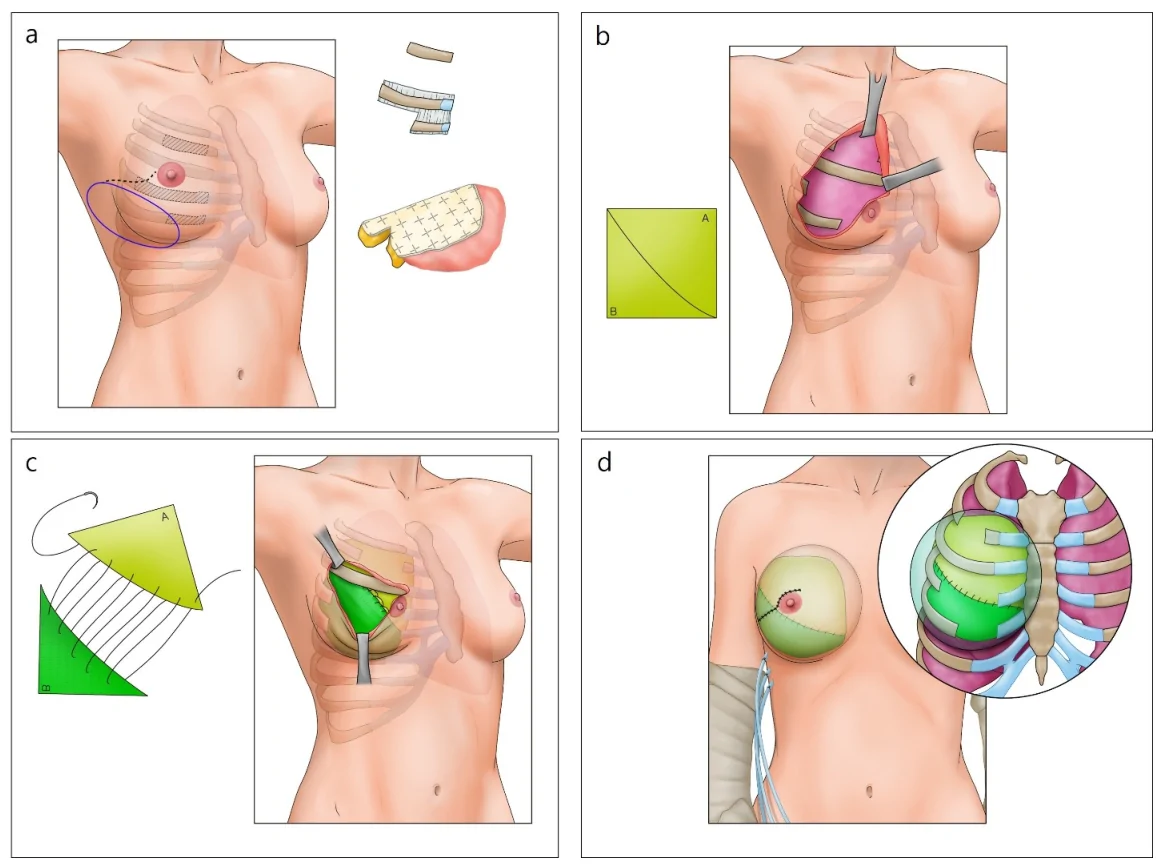
Surgical management of chest-wall recurrence in a malignant phyllodes tumor. (**a**) Local recurrence at the costal cartilage of the second, fourth and fifth ribs after nipple-sparing mastectomy with direct-to-implant (DTI) reconstruction. (**b**) En bloc resection with the involved ribs, opening the pleural cavity and exposing the lung. (**c**) A thick, non-fenestrated acellular dermal matrix (ADM; SCderm, DOF Inc., Hwaseong, Republic of Korea; 20 × 20 cm, 2–3 mm) positioned with its dermal surface toward the implant to seal the pleural cavity, the remainder trimmed and inverted to incorporate into the overlying skin flap. (**d**) Implant reinserted to complete the revisional DTI reconstruction.

After en bloc resection of the involved ribs, the pleura lay widely open with the lung directly visible. The defect was sealed with a single continuous sheet of thick, non-fenestrated acellular dermal matrix (ADM; SCderm, DOF Inc., Hwaseong, Republic of Korea; 20 × 20 cm, 2–3 mm), laid across the defect and anchored to the adjacent rib stumps under tension with heavy interrupted, continuous and horizontal-mattress sutures passed through the matrix and around the ribs, and the implant was reinserted to complete a revisional direct-to-implant reconstruction (Figure 8). The postoperative course was uneventful, with no pneumothorax, persistent air leak or symptomatic effusion.

**Figure 9:**
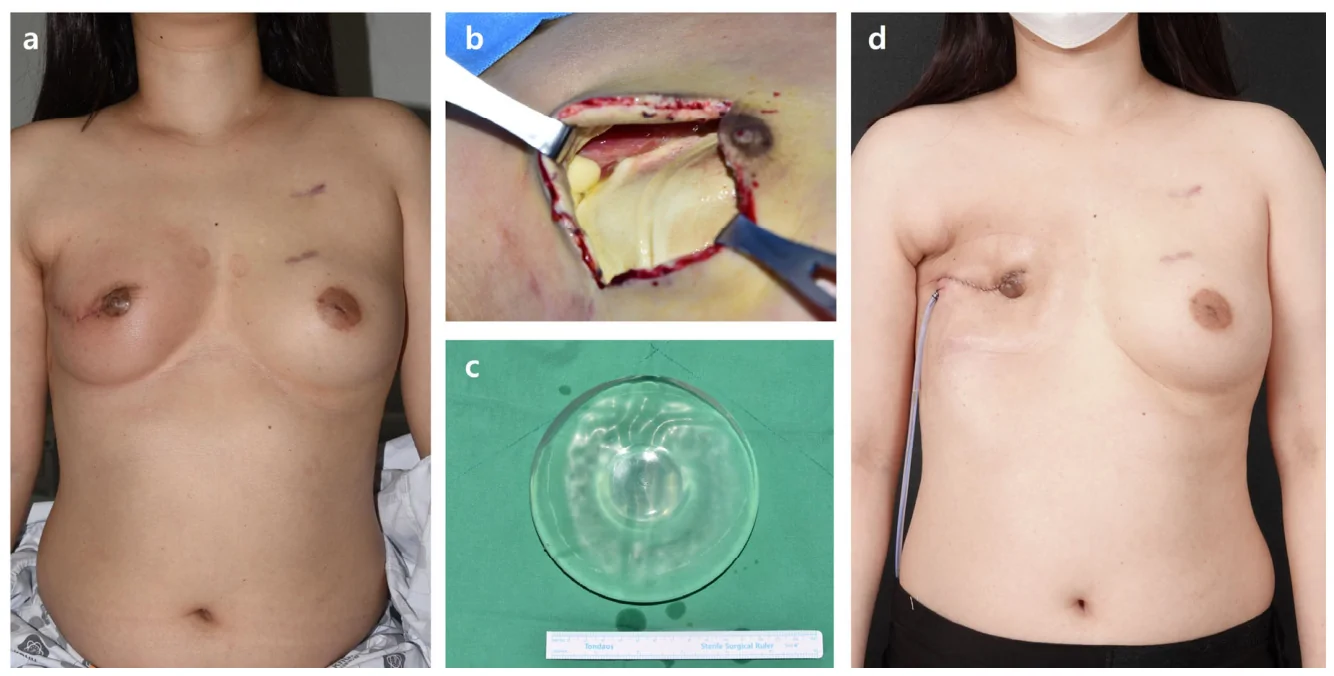
Infected breast implant and its removal, with the acellular dermal matrix (ADM) chest-wall repair preserved. (**a**) Infection of the implant-reconstructed right breast. (**b**) After explantation the ADM remained fully incorporated into the chest wall and overlying skin flap, still sealing the pleural cavity without leakage, providing direct confirmation of matrix integration over an open pleural defect. (**c**) The explanted implant, without rupture. (**d**) Second postoperative day; the patient declined further reconstruction. ADM—acellular dermal matrix.

The revisional reconstruction was initially uncomplicated; however, the overlying breast implant more recently became infected and was explanted; the ADM chest-wall repair remained well incorporated into the chest wall and skin flap, sealing the pleural cavity without leakage, and the explanted implant showed no evidence of rupture (Figure 9). As the patient declined further reconstruction, only a closed-suction drain was placed at explantation, and she has since remained without an implant.

Serial chest computed tomography documenting the thoracic course across the reconstruction is provided as Supplementary Figure S3.

## 4. Discussion

In this single-institution series, most phyllodes tumors were benign (75.1%), with borderline and malignant tumors accounting for 24.9%, a distribution consistent with prior series, in which malignant tumors comprise roughly 20–30% of PTs [5,7]. These findings reinforce a grade-stratified approach in which the extent of surgery escalates with histologic grade (Figure 1). As core needle biopsy reliably assigns histologic grade in only a minority of fibroepithelial lesions, an excisional specimen is often required for definitive grading [4]. This escalation mirrors the prognosis across grades: pooled meta-analyses report local recurrence rates rising from approximately 7–8% in benign tumors to 13–17% in borderline and 18–25% in malignant tumors, and the risk of local recurrence increases significantly from benign to borderline to malignant disease [17,18]. Distant metastasis, by contrast, is essentially confined to the malignant grade, occurring in roughly 20% of malignant tumors and only rarely, if ever, in benign or borderline lesions [5,6,7].

For benign PTs, BCS with negative margins is sufficient, histologic grade and margin status being the principal determinants of local recurrence [2,3]. Although a tumor-free margin of approximately 1 cm is often advised, an ink-negative margin is generally adequate in practice, and re-excision solely to widen a sub-centimeter negative margin is not mandated [14,19]; a large multi-institutional cohort found no further reduction in recurrence with very wide margins, close (<1 mm) margins remaining the main concern [3,20]. Adjuvant radiotherapy improves local control in borderline and malignant disease without a demonstrated survival benefit (hazard ratio 0.43 for local recurrence; absolute risk reduction approximately 10%) [21,22], most consistently after margin-negative BCS and less certainly after mastectomy [18,23,24]. It is therefore best reserved for tumors with adverse features (close or positive margins, large or high-grade tumors, or local recurrence) and is not applied routinely [13].

Borderline and malignant PTs behave more aggressively, with sarcoma-like hematogenous spread in approximately 20% of malignant cases, most often to the lungs and bones [5,6,7]. In a series of seven metastatic malignant PTs, Ramakant et al. observed a uniformly poor outcome and emphasized aggressive primary surgery to minimize local recurrence [7]. Distant metastasis has not been reported in benign PTs and is exceptional in borderline tumors.

For PTs invading the chest wall, full-thickness chest-wall resection with en bloc clearance is required. A recent systematic review of full-thickness chest-wall resection for locally invasive PTs reported feasible short-term local control and symptom relief, although most recurrences were distant and the long-term oncologic benefit remained uncertain [8]. Reconstruction of such defects typically combines rigid support (mesh or titanium) with well-vascularized soft tissue; the LD flap is the most commonly used soft-tissue option, with TRAM, omental, and combined flaps reserved for larger defects [8,9,25,26]. Gupta et al. described staged full-thickness resection with mesh, LD, and omental flap reconstruction for extensive disease [9], and Sandler and Hayes-Jordan summarized the principles of skeletal and soft-tissue chest-wall reconstruction [25]. Biologic meshes such as acellular dermal matrix provide a further reconstructive option in this setting: ADM becomes vascularized and incorporated by the host and shows resistance to infection and adhesion, which makes it attractive if the operative field is potentially contaminated [27,28]. In the present case, a thick, non-fenestrated ADM sutured directly and at multiple points to the rib stumps provided an immediate, watertight seal of a widely opened pleural cavity in which the lung was directly exposed; notably, after the overlying implant later required explantation for infection, the ADM repair was found to have incorporated well into the chest wall and skin flap and continued to maintain pleural integrity, with the underlying lung well aerated on serial computed tomography and respiration preserved both radiographically and symptomatically. This durability, independent of the prosthesis and sustained even under subsequent implant infection, underscores the value of a robustly anchored biologic seal over an exposed pleural defect.

For localized malignant PT without chest-wall invasion, total mastectomy with immediate reconstruction allows oncologic and aesthetic goals to be reconciled (Figure 2). Nipple-sparing mastectomy with implant-based or autologous reconstruction has been reported to be both curative and cosmetically satisfactory if the NAC is uninvolved and the retroareolar margin is negative [10,11,12,13]. Because submuscular implants preserve the ability to monitor for local recurrence, implant-based immediate breast reconstruction is an attractive option in this setting [10,12], whereas LD-based reconstruction provides robust, well-vascularized coverage, particularly in larger defects or if radiotherapy is anticipated [13].

Where full-thickness resection leaves the pleural cavity widely open, the conventional solution is vascularized autologous tissue, prosthetic mesh or matrix being used chiefly to bridge the skeletal defect and usually fenestrated to drape individual rib stumps. In the case presented here, three ribs had been resected and the lung was directly visible, and the defect was sealed instead with a single continuous sheet of thick, non-fenestrated ADM anchored to the rib stumps under tension. A watertight pleural seal was achieved without autologous transfer, and the postoperative course was free of pneumothorax, persistent air leak or symptomatic effusion.

The repair was subsequently inspected under direct vision. When the overlying implant became infected and was explanted, the ADM was found fully incorporated into both the chest wall and the overlying skin flap, still sealing the pleural cavity. Such confirmation is not otherwise obtainable, since a successful repair is never reopened. The finding supports the use of a thick, non-fenestrated matrix as a definitive pleural seal rather than as a skeletal spacer alone, the seal being maintained by biological incorporation. A single case cannot establish this as practice.

Axillary dissection is not routinely indicated given the rarity of nodal involvement, and neither adjuvant chemotherapy nor endocrine therapy has an established role in localized disease [5,8]. In the metastatic setting, chemotherapy is palliative and the prognosis poor, with a reported median overall survival of about 11 months [15,16]. Regimens are sarcoma-directed: ifosfamide-containing combinations achieve the longest progression-free survival, polychemotherapy offers no advantage over single agents, and the number and resectability of metastatic sites remain the dominant prognostic factors [15,16,29]. Systemic chemotherapy is therefore best reserved for metastatic or unresectable disease while complete resection of the primary tumor and, where feasible, of isolated metastases is pursued.

### 4.1. Rationale for the Reconstructive Choices

The dominant reconstructive problem in malignant PT is not volume alone; it is stable, well-vascularized coverage of a large lateral or chest-wall defect, often after previous excision and occasionally in an irradiated field. The latissimus dorsi flap supplies muscle bulk within the arc of rotation of such a defect, tolerates irradiation, and can be raised in the same lateral decubitus position as the mastectomy. Thoracodorsal artery perforator (TDAP) and other fasciocutaneous perforator flaps spare the muscle and are used regularly in our unit for partial breast reconstruction [30]; their limitations, however, become decisive once total reconstruction or chest-wall coverage is required: the pedicle is short, the perforator is a less robust conduit than the thoracodorsal artery itself, and volume must be supplied by skin and fat alone, which is frequently insufficient in a predominantly thin cohort. Raising a TDAP flap also disturbs the thoracodorsal territory and may compromise a subsequent latissimus dorsi flap, removing the most reliable salvage option in a disease with a substantial risk of local recurrence. Where the deficit is superficial and the defect partial, a perforator flap remains a reasonable and less morbid choice.

Direct-to-implant reconstruction was restricted to patients in whom the nipple–areola complex and the skin envelope were uninvolved, the retroareolar margin was negative, the chest wall was intact, and postoperative irradiation was not anticipated; under these conditions nipple-sparing mastectomy with immediate implant reconstruction has been reported to maintain oncologic safety in malignant PT [10,11,12,13]. Where any condition was not met, an autologous option was preferred.

The choice between the extended and the boomerang design followed the geometry of the defect. The boomerang flap (bLD) is a unilateral pedicled design and not a bilateral latissimus dorsi transfer. The extended flap, which harvests the fat overlying the muscle, was used when volume was the limiting requirement and the skin envelope was largely preserved. The boomerang design, in which the skin paddle is drawn as an angled, V-shaped territory, was used when a substantial skin deficit had to be resurfaced as well as filled; it enlarges the cutaneous territory available from a single pedicled flap while permitting a tension-free linear donor-site closure, and so addresses what has historically been the principal limitation of latissimus dorsi reconstruction. The design was reported by our unit for total breast reconstruction [31], has since been evaluated against conventional latissimus dorsi reconstruction with an implant in 85 patients [32], and the vertical variant used here for smaller defects has been described separately [33].

A deep inferior epigastric perforator (DIEP) flap was not used in this series. Free tissue transfer, including DIEP reconstruction, forms part of our routine practice in elective breast reconstruction, and its exclusion here reflects the priorities of this disease rather than any reservation about the technique. Where survival and local control govern, the reconstruction should add as little as possible to operative time, to the number of systems that can fail, and to complications that might delay adjuvant treatment. A pedicled latissimus dorsi flap requires no microvascular anastomosis and no postoperative anticoagulation, whereas a free flap adds a second operative field, a prolonged anesthetic, abdominal donor-site morbidity and a microsurgical failure mode. Breast volume in this population is most often small to moderate, so that a latissimus dorsi flap, combined with an implant where more volume was required, was sufficient; with the boomerang design addressing the skin-territory limitation, the two constraints that would ordinarily favor a DIEP flap were both soluble within a pedicled approach. In a patient with a large breast, ample abdominal volume, an intact chest wall and no urgency about adjuvant treatment, a DIEP flap would be an appropriate alternative.

### 4.2. Generalizability and Institution-Specific Components

The extent to which this experience can be transferred requires comment, since immediate reconstruction presupposes a reconstructive service available at the index operation, and chest-wall disease a combined breast, thoracic and plastic surgical team. Where more than one reconstruction will answer the defect, we prefer the operation with the simpler execution and the lower complication burden, since every additional operative field and failure mode lengthens recovery and may delay adjuvant treatment.

Three tiers should be distinguished. The first comprises recommendations that rest on the biology of the tumor rather than on local resources and are, in our view, broadly applicable: grade-stratified extent of resection, the sufficiency of an intact capsule with ink-negative margins for benign lesions, the avoidance of routine axillary dissection, and the limited role of systemic therapy. The second applies wherever a reconstructive service exists and requires that service at the time of the oncologic operation: immediate mound reconstruction, and the preference for vascularized autologous coverage in an irradiated or previously operated field. The third is institution-specific and is offered as experience rather than as recommendation: the routine availability of full-thickness chest-wall resection with immediate reconstruction, and the particular latissimus dorsi-based repertoire described above.

Where immediate reconstruction is not available, delayed reconstruction after definitive pathology remains a legitimate alternative and does not compromise oncologic outcome; the grade-stratified resection principles of this algorithm apply unchanged in that setting. An institution with a different case mix or different priorities might reasonably select different reconstructive techniques.

As malignant PTs frequently relapse and may progress through repeated local recurrences and successive metastatic sites, the management of recurrent disease warrants explicit consideration. No prospective, disease-specific guideline exists; instead, recurrent and progressive malignant PT is managed pragmatically along soft-tissue sarcoma principles, and every clinical decision is best made within a multidisciplinary tumor board [8,29]. Each recurrence should be confirmed histologically, because grade and morphology can shift between events: benign or borderline lesions may recur as higher-grade or frankly sarcomatous tumors, and the biphasic architecture is often lost at metastatic sites, so a fresh biopsy at relapse informs both prognosis and subsequent therapy [2,7,29]. For an isolated locoregional recurrence without distant disease, complete re-resection to negative margins remains the treatment of choice, with chest-wall resection and reconstruction if the recurrence invades the thoracic wall; radiotherapy is added for close or positive margins or after repeated relapse if not previously delivered [8,21,29]. In the metastatic and re-recurrent setting, surgery retains a central role: resection of oligometastatic or isolated, resectable metastases (most often pulmonary) is pursued where feasible, because resectability of metastases is among the dominant prognostic factors, whereas systemic therapy is reserved for unresectable, multifocal, or rapidly progressive disease [15,16,29]. First-line systemic treatment follows sarcoma-directed regimens (anthracycline- and/or ifosfamide-based), and after progression there is no established standard; later-line choices are extrapolated from soft-tissue sarcoma practice, and enrollment in clinical trials and molecularly guided therapy should be considered for chemotherapy-refractory patients [15,16,29,34,35].

These limited systemic options have prompted growing interest in the molecular characterization of malignant PT, and next-generation sequencing (NGS) has begun to define a genomic landscape that is distinct from common breast carcinoma and more closely resembles that of soft-tissue sarcoma. Early targeted sequencing identified recurrent MED12 (G44 hotspot) mutations across all histologic grades, an event shared with fibroadenoma and therefore likely early in tumorigenesis, whereas loss-of-function alterations in TP53, RB1, and NF1, together with high-level copy-number changes, were largely confined to malignant tumors; that work also flagged potentially actionable IGF1R and EGFR amplifications [36]. Subsequent comprehensive profiling has refined this picture: TERT promoter mutations (~70%), MED12 (~50–60%), and TP53 (~50%) are the most frequent alterations, with less common, potentially targetable events in the receptor tyrosine kinase/RAS (NF1, EGFR, BRAF) and PI3K/AKT/mTOR (PIK3CA) pathways, and TERT promoter mutations in particular appear to increase in frequency from benign to malignant-grade, suggesting a role in progression [37,38]. Importantly, occasional actionable gene fusions occur: a TPM4–NTRK1 fusion identified by RNA-based sequencing was associated with a durable clinical response to the TRK inhibitor larotrectinib, illustrating the value of including fusion detection in the diagnostic workup of advanced disease [37]. Immunotherapeutic biomarkers are generally modest: PD-L1 positivity is seen in roughly 15% of cases, although isolated responses to immune-checkpoint blockade have been reported in tumors with a high tumor mutational burden [37,39]. Registry data linking genomic profiles to chemotherapy outcomes further suggest that alterations such as CDKN2A or TERT may track with treatment resistance, though these associations remain exploratory [39]. Taken together, current evidence supports obtaining NGS, ideally with RNA-based fusion analysis, in patients with metastatic, recurrent, or chemotherapy-refractory malignant PT, both to identify candidates for genotype-matched therapy or clinical trials and to advance the still-incomplete understanding of this rare tumor; no targeted agent has been validated prospectively in malignant PT, and such testing should complement rather than replace complete surgical resection [36,37,38,39].

The recurrent tumor in the present case fits this landscape without adding an actionable target. Sequencing (TruSight Oncology 500 with HRD, 523 genes; Illumina NextSeq 550Dx) showed a MED12 exon 2 mutation (p.Gly44Ser), the characteristic fibroepithelial driver, together with oncogenic TP53 (p.Arg213Pro) and NRAS (p.Gly13Arg) mutations and a chromosome 8q gain. The tumor mutational burden was low (2.3 mutations/Mb), the tumor was microsatellite stable, and no tier-1 alteration was identified. Local control by resection and reconstruction therefore remained the only intervention with a demonstrable effect on outcome.

This study has several limitations. The reconstructive repertoire reported here reflects a specialized single-center practice in which a reconstructive service is available at the index operation, and the reconstructive components of the algorithm should be read in that light. First, it is a retrospective, single-institution review and is therefore subject to selection and information bias. Second, the charted cohort included indeterminate needle-biopsy impressions that could not be definitively graded, and local recurrence, mortality, and systemic therapy were summarized descriptively (Table 4); these analyses are exploratory: the small number of events, the short and variable follow-up, and confounding by indication, particularly for adjuvant radiotherapy and chemotherapy, which were preferentially given to malignant tumors, preclude reliable causal inference. The treatment approach is therefore presented as a descriptive institutional algorithm rather than a comparative outcome study. Third, no systematic molecular or genomic profiling was undertaken across the cohort, sequencing having been performed only for the single recurrent tumor described in Section 3.5, so the discussion of next-generation sequencing and targeted therapy reflects external evidence and not systematic institutional data. Finally, the rarity of malignant phyllodes tumors limited the number of total-mastectomy, reconstructive, and irradiated cases, precluding formal subgroup comparison and statistical inference. A further limitation concerns the composition of the surgical cohort itself. Because breast-conserving surgery and partial mastectomy are used synonymously in most centers, the cohort could not be assembled from recorded procedure names; every operative record in the charted cohort was therefore re-audited for whether the resection defect required a reconstructive procedure, and four resections recorded as partial mastectomy and closed directly were counted with the conserving group. The audit was applied to the whole charted cohort against the operative record, the histopathological report and the follow-up file, and no case remained ambiguous; the criterion is nonetheless an operative judgment and not a coded variable, and any external validation would need to reconstruct it from operative criteria instead of from procedure names.

## 5. Conclusions

Phyllodes tumors require a grade-stratified surgical strategy that balances oncologic safety against preservation of breast form. Benign tumors are effectively managed by BCS, whereas borderline and malignant tumors warrant wider resection or total mastectomy, with immediate reconstruction offering a means to restore the breast after total mastectomy. Given the sarcoma-like behavior of high-grade tumors and their potential for chest-wall recurrence and distant metastasis, multidisciplinary management and long-term surveillance remain essential.

## Figures and Tables

**Table 1:** Classification, demographics, and surgical management of the definitively graded phyllodes tumor cohort across histologic grades (n = 334).

Characteristic	Benign (n = 251)	Borderline (n = 55)	Malignant (n = 28)	Total (n = 334)	*p* Value
***Histologic classification***					
No. of patients, n (%)	251 (75.1)	55 (16.5)	28 (8.4)	334 (100)	NA
***Demographics***					
Age, yr, mean ± SD ^a^	40.4 ± 10.4	50.0 ± 13.3	48.0 ± 14.1	46.8 ± 13.2	0.017 ^b^
BMI, kg/m^2^, mean ± SD ^a^	22.1 ± 5.0	23.9 ± 5.2	22.5 ± 3.0	23.0 ± 4.6	0.411 ^b^
***Surgical management, n***					
Breast-conserving surgery (BCS), direct closure	247	48	13	308	
Oncoplastic breast-conserving surgery	1	3	3	7	
Total mastectomy	3	4	12	19	<0.001 ^c^

Classification percentages are provided for the 334 definitively graded phyllodes tumors; borderline and malignant (non-benign) tumors together accounted for 83 (24.9%). Of 417 patients charted as having a “phyllodes tumor,” 16 non-phyllodes lesions were excluded, and a further 67 lesions with an indeterminate needle-biopsy impression or no stated grade were excluded from the graded analysis; none of the latter underwent mastectomy. Breast-conserving surgery with direct closure denotes a conserving resection closed directly; oncoplastic breast-conserving surgery denotes a conserving resection reconstructed by the plastic surgical team (Section 2.1). The surgical cohort of Table 2, Table 3 and Table 4 comprises the latter together with all total mastectomies (n = 26). BMI—body mass index; SD—standard deviation. ^a^ Age and body mass index were retrievable for 80 and 54 patients, respectively; the Total column gives the pooled mean ± SD across grades. ^b^ One-way analysis of variance across benign, borderline, and malignant tumors. ^c^ Fisher’s exact test for total mastectomy (malignant vs. benign or borderline); mastectomy, and total mastectomy in particular, was more frequent for malignant-grade. The surgical characteristics and outcomes of the surgical cohort are detailed in Table 2, Table 3 and Table 4.

**Table 2:** Patient, tumor, and treatment characteristics of the surgical cohort (n = 26).

Characteristic	n = 26
***Patient and tumor***	
Age, yr, median (range) ^a^	47.5 (20–89)
Tumor size, cm, median (range) ^a^	6.7 (1.1–27.0)
Histologic grade, n (%)	
Benign	4 (15.4)
Borderline	7 (26.9)
Malignant	15 (57.7)
Multifocal, n (%)	7 (26.9)
Skin involvement, n (%)	5 (19.2)
Chest-wall involvement, n (%)	3 (11.5)
Lymph-node metastasis, n (%)	1 (3.8)
***Breast*** ***Surgery***	
Total mastectomy, n (%)	19 (73.1)
Oncoplastic BCS, n (%) ^b^	7 (26.9)
***Breast*** ***Reconstruction***	
Immediate reconstruction, n (%)	21 (80.8)
Breast mound reconstruction, n (%)	14 (53.8)
LD-based flap	8
Direct-to-implant (DTI)	6
Chest-wall/soft-tissue coverage, n (%)	7 (26.9)
Lateral thoracodorsal flap ± STSG	3
Local flap (advancement or rotation flap)	3
LD flap	1
Primary closure, n (%)	5 (19.2)
***Adjuvant/systemic therapy***	
Phyllodes-directed radiotherapy, n (%)	7 (26.9)
Systemic chemotherapy, n (%)	6 (23.1)
Doxorubicin (A)	3 (11.5)
Doxorubicin + ifosfamide (AI)	3 (11.5)
Chemotherapy duration, mo, median (range)	2.3 (1.7–9.5)
***Follow-up***	
Median follow-up, mo (range)	32.7 (0.1–99.5)

All percentages are of the 26 patients of the surgical cohort. Reconstructive procedures are listed by reconstructive intent and are not pooled. LD-based flaps comprised extended, vertical and boomerang designs; local flaps comprised advancement, rotation and external oblique myocutaneous flaps. A—doxorubicin; AI—doxorubicin plus ifosfamide; DTI—direct-to-implant; LD—latissimus dorsi; LTD—lateral thoracodorsal; STSG—split-thickness skin graft. ^a^ Age was recorded for 22 of the patients (median 47.5, range 20–89 years); tumor size, reported as the maximum diameter, was available for all 26. ^b^ Breast-conserving resections reconstructed by the plastic surgical team; conserving resections closed directly are not part of this cohort. Skin involvement, chest-wall involvement, and lymph-node metastasis were each confined to malignant tumors.

**Table 3:** Adjuvant radiotherapy in the surgical cohort (n = 26).

Phyllodes-Directed Radiotherapy Course Detail (n = 6)	
RT field	Chest wall/reconstructed breast only, 3; chest wall/reconstructed breast + regional nodal, 2; reconstructed breast only, 1
Fractionation	Conventional, 4; hypofractionated, 2
Tumor-bed boost	Yes, 3; No, 3
Total dose, Gy, median (range)	52.6 (50.0–60.0)

Radiotherapy directed at the phyllodes tumor was confined to malignant disease (7 of 15 malignant tumors [46.7%] vs. 0 of 11 benign or borderline; Fisher’s exact *p* = 0.010). Of 26 patients, 7 were irradiated and 19 were not. Course detail is shown for 6 of the 7 irradiated patients; one course was delivered at an outside hospital and its field, fractionation, and boost details were unavailable. RT—radiotherapy.

**Table 4:** Univariable analysis of oncologic outcomes in the surgical cohort (n = 26).

Factor	Level	Local Recurrence, n/N (%)	Disease-Related Death, n/N (%)	*p* Value ^a^
Histologic grade	Benign	0/4 (0)	0/4 (0)	
	Borderline	1/7 (14.3)	0/7 (0)	
	Malignant	3/15 (20.0)	2/15 (13.3)	0.614
Extent of surgery	Total mastectomy	2/19 (10.5)	NA	
	Oncoplastic BCS	2/7 (28.6)	NA	0.287
Adjuvant radiotherapy	No	2/19 (10.5)	NA	
	Yes	2/7 (28.6)	NA	0.287
Adjuvant chemotherapy	No	1/20 (5.0)	NA	
	Yes	3/6 (50.0)	NA	0.028 ^b^

^a^ *p* values are from two-sided Fisher’s exact tests for local recurrence and are exploratory given the small number of events (4 recurrences). Disease-related death is shown for the histologic-grade stratum only; both deaths occurred in malignant tumors, and NA marks strata that are not applicable to the death comparison. ^b^ Odds ratio for local recurrence with chemotherapy, 19.0. Chemotherapy was administered only to patients with malignant, more advanced, or metastatic disease, so receipt of chemotherapy is a marker of high-risk disease rather than a cause of recurrence; this association reflects confounding by indication and must not be interpreted as a harmful or ineffective treatment effect.

## Data Availability

The datasets generated and analyzed during the current study are not publicly available owing to institutional and patient-privacy restrictions; they are available from the corresponding author on reasonable request.

## Supplementary Materials

The following supporting information can be downloaded at: https://www.mdpi.com/article/10.3390/jcm15187226/s1, Figure S1: Restaging fused PET/CT of the isolated chest-wall recurrence; Figure S2: Histopathological findings of the recurrent malignant phyllodes tumor in the rib; Figure S3: Serial axial chest computed tomography across the chest-wall resection and its subsequent course.
