# Supplementary material for: Management of Breast Phyllodes Tumors: A Grade-Stratified Surgical and Reconstructive Strategy
Source: J Clin Med. 2026 Sep 17;15(18):7226. doi: 10.3390/jcm15187226 (PMC13607788; doi:10.3390/jcm15187226)
Supplement: Supplementary file 1 [file jcm-15-07226-s001.zip › jcm-4454767-supplementary.pdf]

Supplementary Materials

Management of Breast Phyllodes Tumors: A Grade-Stratified Surgical and Reconstructive Strategy

Manuscript ID: jcm-4454767 · Journal of Clinical Medicine

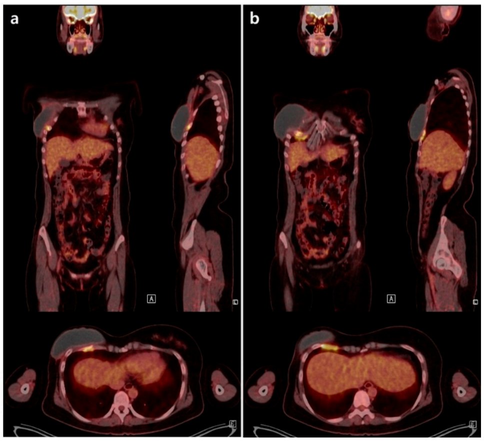

Figure S1. Restaging fused PET/CT of the isolated chest-wall recurrence. Intense hypermetabolic uptake localizes to the costal cartilage of the second, fourth, and fifth ribs, confirming an isolated chest-wall recurrence with rib involvement and no distant disease. (a,b) Coronal, sagittal, and axial images from the same study at two different rib levels, shown to demonstrate the location of each involved segment. PET/CT, positron emission tomography–computed tomography.

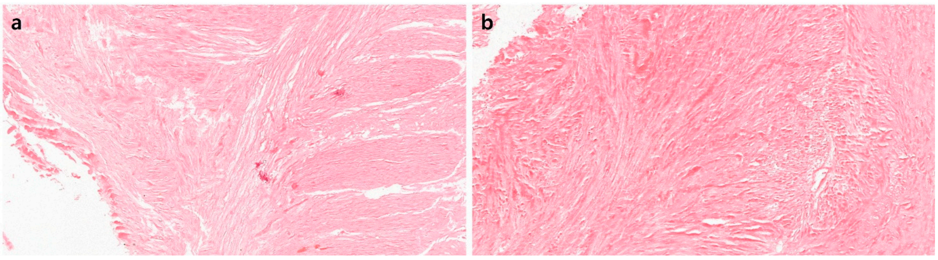

Figure S2. Histopathological findings of the recurrent malignant phyllodes tumor in the rib (hematoxylin and eosin stain). (a) Low-power and (b) higher-power views (original magnification, ×20 and ×40, respectively) show marked stromal hypercellularity with a dense, fascicular spindle-cell proliferation and nuclear atypia. The tumor exhibits an infiltrative growth pattern into the adjacent costal bone and soft tissue, with complete loss of the typical biphasic leaf-like architecture, replaced by a purely sarcomatous appearance.

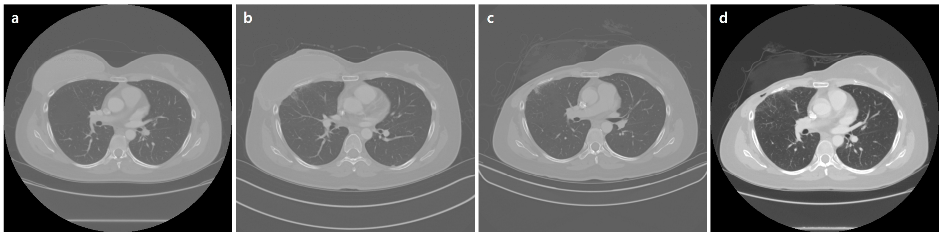

Figure S3. Serial axial chest computed tomography (lung window) across the chest-wall resection and its subsequent course. (a) Preoperative study obtained 2.8 months before en bloc chest-wall resection

and reconstruction with a thick, non-fenestrated acellular dermal matrix (ADM). (b) Study obtained 1.6 months after reconstruction and one day before explantation of the overlying implant for infection. (c,d) Studies obtained 1.2 and 2.2 months after explantation. No pneumothorax, persistent air leak, or symptomatic pleural effusion is seen at any postoperative time point, and the reconstructed chest wall remains stable throughout, including after removal of the implant. ADM, acellular dermal matrix.
